# Diagnostic Accuracy of Ultrasound and MRI in the Mapping of Deep Pelvic Endometriosis Using the International Deep Endometriosis Analysis (IDEA) Consensus

**DOI:** 10.1155/2020/3583989

**Published:** 2020-01-30

**Authors:** T. Indrielle-Kelly, F. Frühauf, M. Fanta, A. Burgetova, D. Lavu, P. Dundr, D. Cibula, D. Fischerova

**Affiliations:** ^1^First Faculty of Medicine, Charles University in Prague, Czech Republic; ^2^Department of Obstetrics and Gynecology, Burton Hospitals NHS, Belvedere Road, Burton-on-Trent DE13 0RB, West Midlands, UK; ^3^Department of Obstetrics and Gynecology, First Faculty of Medicine, Charles University and General University Hospital in Prague, 128 08 Apolinářská 18, Czech Republic; ^4^Department of Radiology, First Faculty of Medicine, Charles University and General University Hospital in Prague, Studničkova 2, 128 00 Prague, Czech Republic; ^5^ACALM Study Unit, Birmingham, UK; ^6^Department of Pathology, First Faculty of Medicine, Charles University and General University Hospital in Prague, U Nemocnice 499, 128 08 Prague, Czech Republic

## Abstract

**Objectives:**

The primary aim was to investigate the diagnostic accuracy of transvaginal ultrasound (TVS) and magnetic resonance imaging (MRI) in the mapping of deep pelvic endometriosis (DE) in a diseased population. The secondary aim was to offer first insights into the clinical applicability of the new International Deep Endometriosis Analysis group (IDEA) consensus for sonographic evaluation, which was also adapted for MRI and surgical reporting in this study.

**Methods:**

The study was a prospective observational cohort study. In this study, consecutive women planned for surgical treatment for DE underwent preoperative mapping of pelvic disease using TVS and MRI (index tests). The results were compared against the intraoperative findings with histopathological confirmation (reference standard). In case of disagreement between intraoperative and pathology findings, the latter was prioritised. Index tests and surgical findings were reported using a standardised protocol based on the IDEA consensus.

**Results:**

The study ran from 07/2016 to 02/2018. One-hundred and eleven women were approached, but 60 declined participation. Out of the 51 initially recruited women, two were excluded due to the missing reference standard. Both methods (TVS and MRI) had the same sensitivity and specificity in the detection of DE in the upper rectum (UpR) and rectosigmoid (RS) (UpR TVS and MRI sensitivity and specificity 100%; RS TVS and MRI sensitivity 94%; TVS and MRI specificity 84%). In the assessment of DE in the bladder (Bl), uterosacral ligaments (USL), vagina (V), rectovaginal septum (RVS), and overall pelvis (P), TVS had marginally higher specificity but lower sensitivity than MRI (Bl TVS sensitivity 89%, specificity 100%, MRI sensitivity 100%, specificity 95%; USL TVS sensitivity 74%, specificity 67%, MRI sensitivity 94%, specificity 60%; V TVS sensitivity 55%, specificity 100%, MRI sensitivity 73%, specificity 95%; RVS TVS sensitivity 67%, specificity 100%, MRI sensitivity 83%, specificity 93%; P TVS sensitivity 78%, specificity 97%, MRI sensitivity 91%, specificity 91%). No significant differences in diagnostic accuracy between TVS and MRI were observed except USL assessment (*p*=0.04) where MRI was significantly better and pouch of Douglas obliteration (*p*=0.04) where MRI was significantly better and pouch of Douglas obliteration (*κ*) = 0.727 [*p*=0.04) where MRI was significantly better and pouch of Douglas obliteration (*κ*) = 0.727 [*p*=0.04) where MRI was significantly better and pouch of Douglas obliteration (*p*=0.04) where MRI was significantly better and pouch of Douglas obliteration (

**Conclusion:**

We found that both imaging techniques had overall good agreement with the reference standard in the detection of deep pelvic endometriosis. This is the first study to date involving the IDEA consensus for ultrasound, its modified version for MRI, and intraoperative reporting of deep pelvic endometriosis in clinical practice.

## 1. Introduction

Endometriosis has been recognized for decades as the leading cause of pelvic pain in women of reproductive age [1]. Cornille et al. defined deep endometriosis (DE) as infiltration of the tissue deeper than 5 mm with a typical location in the wall of bladder and bowel, uterosacral ligaments (USL), vagina, and rectovaginal septum (RVS) [2]. Transvaginal ultrasound (TVS) and magnetic resonance imaging (MRI) are often used for preoperative staging of the disease with high accuracy. In the most recent meta-analysis of these methods by Guerriero et al., both showed similar performance when assessing DE in the rectosigmoid, uterosacral ligaments, and rectovaginal septum [3]. Studies comparing TVS and MRI in the assessment of bladder DE are scarce, and the best available evidence for TVS shows sensitivity 62% and specificity 100% [4], which was similar to MRI performance noted in a different systematic review (sensitivity 64%, specificity 98%) [5]. According to the Cochrane review by Nisenblat et al. [6], TVS and MRI are accurate in diagnosing endometriomas and based on a limited evidence also lesions in the lower bowel. Ultrasound could be more useful in identifying pelvic DE compared with MRI, but none of the imaging methods could be suggested to replace surgical staging of overall pelvic endometriosis.

Patients with advanced disease ideally ought be treated in an endometriosis center, which should have available advanced imaging with expert image readers although this is not always stated as a compulsory requirement for the endometriosis center accreditation [7]. Centres choose their imaging of choice mostly based on the available expertise, which frequently tend to be MRI. However, ultrasound has many advantages over MRI, starting with no known contraindications or need for patient preparation (starving, etc.). It is also cheaper, less time-consuming, and due to the dynamic aspects of ultrasound useful in the assessment of adhesions and site-specific tenderness.

In 2016, the International Deep Endometriosis Analysis group (IDEA) published a consensus opinion with an aim to standardise the nomenclature of ultrasound-based endometriosis evaluation [8]. In 2017, the European Society for Urogenital Radiology (ESUR) published guidelines on technical protocol for MRI assessment of pelvic DE [9]. This study's primary aim was to investigate the diagnostic accuracy in pelvic DE mapping of two common imaging modalities (TVS and MRI) in a diseased population. The secondary aim was to offer first insights into the clinical applicability of the IDEA consensus and also its use for MRI and surgical reporting.

## 2. Methods

### 2.1. Study Design and Data Collection

This was a prospective observational cohort study led by a clinical protocol based on the IDEA consensus [10], reporting the diagnostic accuracy of TVS and MRI when mapping pelvic DE. Prior to starting this real-world study, we have drafted the study design following the Standards for Reporting of Diagnostic Accuracy (STARD) guidelines [11]. To compare the diagnostic performance of both TVS and MRI in the mapping of pelvic DE, participants underwent two index tests (TVS and MRI), which were reported using a predefined protocol based on the ultrasound IDEA consensus [8] with its modified version for MRI evaluation (Supplementary [Supplementary-material supplementary-material-1]). Results from the index tests were compared against intraoperative findings with histopathological confirmation where available as the reference standard, which was reported using the same evaluation protocol based on the IDEA consensus.

Sonographers and radiologists were blind to any previous imaging and clinical examination findings, surgeons had access to all reports in order to plan the surgery and multidisciplinary team, and histopathologists were blind to the imaging results but not to the operative findings.

### 2.2. Participants

As our study was aimed at detecting the accuracy of imaging modalities in the mapping of DE, we required participants in whom surgical treatment was planned for high suspicion of DE, hence recruiting them from patients in an endometriosis center. Based on the previous literature with a similar design [12], the sample size at 95% confidence level was calculated to be 44 (using the highest value based on individual anatomical site prevalences of DE). All patients with suspected pelvic DE planned for surgical treatment were consecutively approached to participate in the study. DE was suspected on the basis of (1) clinical symptoms and physical examination and/or (2) nonexpert ultrasound findings and/or (3) previous operative findings from the referring institution (diagnostic laparoscopies without surgical treatment). To be eligible to participate, the women had to be of reproductive age (18–55 years) with index tests and operation performed within four months of recruitment. A maximum four-month interval was established as the longest reasonable time frame based on the departmental case flow. No changes to hormonal treatment were allowed during this period to avoid influencing findings [13]. Participants were excluded if they had one or no index test or their index tests revealed findings suspicious of malignancy. Those with absent reference standard and those whose index-test-to-operation time interval exceeded 4 months were also excluded.

### 2.3. Index Tests

As a part of the preoperative assessment, each participant underwent TVS and TAS by one of two expert gynecologists with >10 years of experience in pelvic and abdominal ultrasonography (level 3 as defined by EFSUMB [14]) and MRI scan was interpreted by an experienced radiologist with >10 years of experience in gynecologic and abdominal imaging; all followed the predefined protocol based on the IDEA consensus [8].

In addition to the original IDEA consensus, assessment of pelvic portion of the ureter was included in the evaluation protocols with a dilatation cutoff value >3 mm [15] for identification of obstruction (hydroureter) due to DE (intrinsic or extrinsic). Renal dilatation (hydronephrosis) was graded as grade 1 (distended renal sinus), grade 2 (distended renal pelvis and calyces), and grade 3 (sacciform hydronephrosis with renal parenchyma atrophy) [16]. IDEA consensus was adapted to MRI with the following modifications in soft markers evaluation: omitting site-specific tenderness, replacing sliding sign by “sign of adhesions” (Supplementary [Supplementary-material supplementary-material-1]). Technical parameters and settings of the index tests are listed in Table 1.

### 2.4. Reference Standard

To reflect the current practice [17], visual confirmation from laparoscopy was used as a reference standard along with histopathological confirmation for every participant but not necessarily for every possible DE site. When the anatomical site showed no signs of DE on laparoscopy, findings were recorded as no DE and biopsy was omitted. When DE was confirmed visually, biopsy or resection was performed for histopathological evaluation. Laparoscopic evaluation followed standard steps as outlined by ESHRE guidelines [17]. All participants underwent a surgical procedure performed by one of the two gynecologists with >10 years of experience in advanced laparoscopy who were assisted by an urologist and/or colorectal surgeon where indicated. The preferred approach was laparoscopic, aiming to remove all or majority of the disease from affected areas by various techniques including shaving, discoid, and segmental resections. Intraoperative findings were described using the same evaluation protocol based on the IDEA terminology and definitions, describing all sites of possible pelvic DE. Histology examination was recorded as negative when it failed to identify the typical endometriosis features (glands and stroma) and when in dispute, pathology findings were prioritised over laparoscopy findings.

### 2.5. Statistical Analysis

Data were recorded as binary sets, and statistical analysis was carried out using SPSS. The sensitivity and specificity with their corresponding 95% confidence intervals (CI), positive and negative predictive values (PPV and NPV, respectively), and positive and negative likelihood ratios (LR+ and LR−, respectively) were calculated for the index tests across various locations of the pelvic disease. The diagnostic performance of both methods was compared using McNemar's test and a probability value (*p* value) <0.05 was regarded significant. Cohen's kappa value (*κ*) was used to determine the level of agreement between the index tests and reference standards regarding the presence or absence of DE lesions in all individual areas of the pelvis. Agreement was interpreted based on guidelines by Altman [18]: *κ* < 0.20, poor agreement; 0.21–0.40, fair agreement; 0.41–0.60, moderate agreement; 0.61–0.80, good agreement; 0.81–1.00, very good agreement.

### 2.6. Ethical Approval

The local ethics committee approved the study protocol, and informed consent was obtained from all subjects (study number 1249/16 S-IV, approved version 1486/16 IS).

## 3. Results

This study was conducted following the publication of IDEA consensus and ethical approval of the study, from August 2016 to February 2018.

## 4. Participants

**Figure 1:**
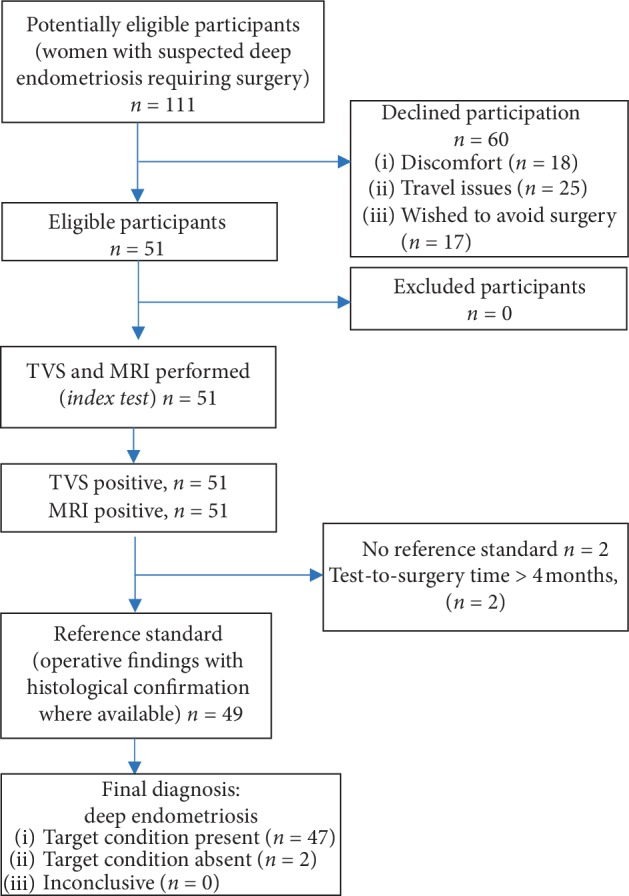
STARD Flow diagram. *N*; number of participants; TVS, transvaginal ultrasound; MRI, magnetic resonance imaging.

Out of 111 patients who were approached, 51 women agreed to participate in the study and underwent index tests. Of the women who were approached, 60 declined participation due to unwillingness to undergo two imaging tests, struggle with multiple appointments, or they opted to avoid surgery. These were the main reasons why women chose to avoid the second imaging (in most cases MRI). Two out of 51 enrolled participants delayed surgery for reproductive reasons hence were excluded from the study due to the absent reference standard (Figure 1). The final analysis was based on the data from 49 participants. All participants were Caucasian and of similar socioeconomic background. Further participant demographics are listed in Table 2.

The average interval between the first index test and the operation was 41 days (3–118 days). The presence of DE was visually and histologically confirmed in 95.9% of cases (47/49) (Table 2). The 2 disease-free cases had only adhesions without visual and histological confirmation of DE. Forty-seven participants (95.9%) underwent laparoscopic procedure, and two (4.1%) had laparoscopy with a conversion to laparotomy due to difficult laparoscopic surgery. The prevalence of histologically confirmed DE lesions across the various sites among the participant population including uterosacral ligaments 69.4% (34/49), rectosigmoid 34.7% (17/49), vagina 22.4% (11/49), upper rectum 20.4% (10/49), bladder 18.4% (9/49), and rectovaginal septum 12.2.% (6/49). Pouch of Douglas obliteration was present in 81.6% (40/49) and hydroureter due to extrinsic DE was in 10.2% (5/49). We recorded 3 areas in 2 patients with visually described DE on laparoscopy, where histology failed to identify endometrial glands and stroma, and hence reference standard was recorded as negative (involvement of uterosacral ligaments and rectosigmoid).

## 5. Test Results

**Figure 2:**
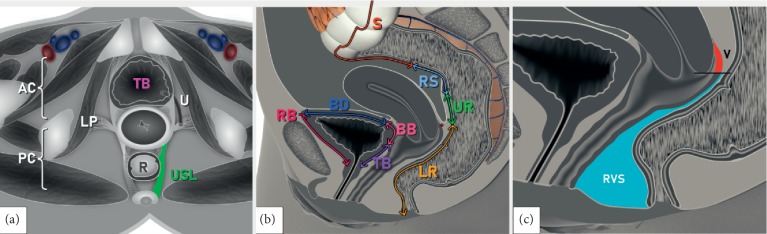
Schematic drawings demonstrating the IDEA proposed anatomical definitions of deep pelvic endometriotic lesions [7]: (a) pelvis—transverse plane at the level of cervix; (b) pelvis—longitudinal plane showing bowel and bladder sections, (c) pelvis—longitudinal plane demonstrating vagina and adjacent structures. AC, anterior compartment; BB, bladder base; BD, bladder dome; LP, lateral parametria; LR, lower rectum; PC, posterior compartment; RB, retroperitoneal part of the bladder; R; rectum; RS, rectosigmoid; RVS, rectovaginal septum; S; sigmoid; TB, trigonum (bladder); U; ureter; UR, upper rectum; USL, uterosacral ligament; V; vagina.

Results of the index tests in comparison with the reference standard are detailed in Table 3, according to the anatomical areas of the DE lesion location (Figure 2, Supplementary [Supplementary-material supplementary-material-1]). All participants underwent both TVS and TAS although all cases of hydroureter in our cohort were identified on TVS. Ultrasound and MRI performances were not statistically different except in the assessment of pouch of Douglas (POD) obliteration where TVS was superior to MRI (*p*=0.040) and in the uterosacral ligaments (USLs) where MRI was better in detecting DE in the USL in general (*p*=0.039). The difference in the detection of DE in USL was only present on the right side (*p* = 0.001) with the left side showing no difference (*p* = 0.220). On using Cohen's *κ*, we found good agreement between both TVS and reference standards *κ* = 0.727 (*p* ≤ 0.001) and MRI and reference standards, *κ* = 0.755 (*p* ≤ 0.001).

Please note these are only schematic drawings of various anatomical sites described in the IDEA consensus [8].

## 6. Discussion

**Figure 3:**
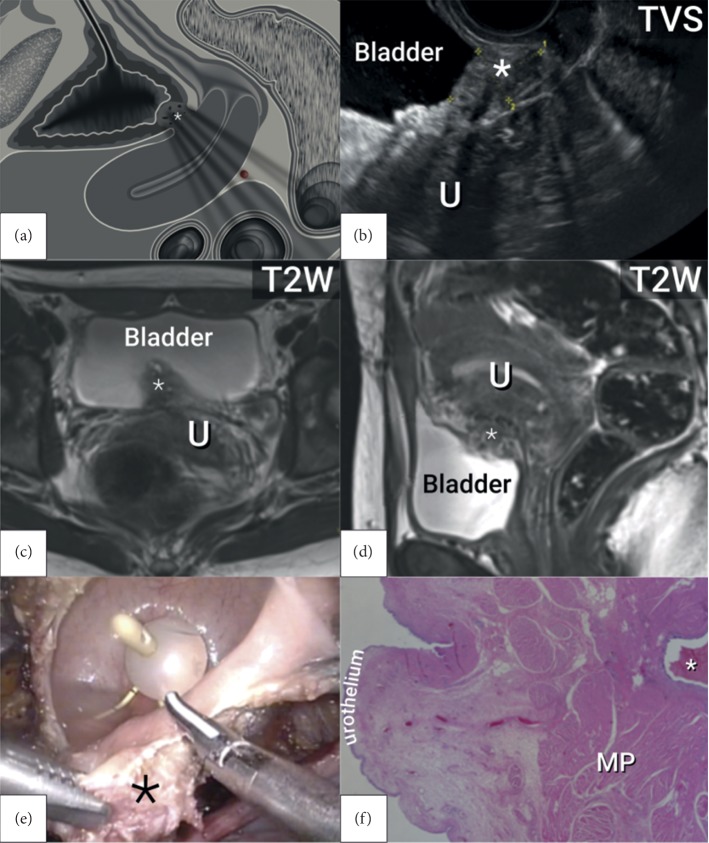
Nodule of deep endometriosis in the bladder base: (a) schematic drawing in the longitudinal view; (b) transvaginal ultrasound in the longitudinal plane; (c) magnetic resonance imaging in the transverse plane; (d) magnetic resonance imaging in the longitudinal plane; (e) laparoscopic resection; (f) histology. MP, muscularis propria; TVS, transvaginal ultrasound; T2W, T2-weighted magnetic resonance imaging; U; uterus; ^*∗*^, deep endometriosis lesion.

Accurate mapping of DE is essential in preoperative planning in order to consent patient**s** adequately and organises a multidisciplinary team and the estimated theatre time. Our primary aim was to assess the diagnostic accuracy of TVS and MRI in preoperative pelvic DE mapping on the same cohort, using one standardised protocol for index tests and reference standard. We found that TVS and MRI were similar in their performance in endometriosis mapping; only in the assessment of USLs did MRI achieve significantly better results (*p*=0.039); and in POD obliteration, TVS showed significantly higher accuracy (*p*=0.040) (Figure 3). The unique aspect of our study is the adaptation of the only international imaging consensus on endometriosis, which was originally intended for ultrasound assessment, for use in MRI and intraoperative reporting of DE.

There are no studies, which compared TVS and MRI in DE mapping on the same cohort by using the same standardised protocol among index tests and reference. Our study's prospective design and standardised protocol based on the IDEA consensus which together with a high-end imaging technology used by expert sonographers and radiologists and experienced surgeons in a referral center setting offer valid data on the mapping capability of the investigated methods.

One of the limitations of this study was a poor participant uptake, where patients declined participation because of discomfort, difficulty attending multiple appointments, or they wished to avoid surgery. Although the sample size was counted as adequate, it resulted in low or zero incidence of certain lesions. It could be also argued that the high prevalence of DE (95.9%) amongst our participants indicates selection bias, but our study does not examine the diagnostic ability of TVS and MRI to detect disease from the general population. It investigates the ability of the imaging modalities to accurately map the disease in a diseased population and as such resembles the previously published studies [12, 19]. Indeed, our aim was to assess the diagnostic accuracy of imaging methods in individual anatomical sites; therefore, our inclusion criteria limited recruitment only to women very likely having the disease. Another limitation is a missing pathological confirmation of the disease in some sites. In our cohort, only 24.5% of cases (12/49) had a discoid or segmental resection with full pathological evaluation, and although the disease was histologically confirmed in 95.9% patients (47/49), it was not available for every anatomical site of potential DE. A biopsy sample can miss foci of endometriosis, which might only be found if the entire organ was removed. As pathology was prioritised over laparoscopy findings as reference standard in this study, there were 2 participants (4.1%) with obvious clinical DE but negative histology (2 uterosacral ligaments and 1 rectosigmoid involvement). Pathology examination was recorded as negative when it failed to identify the typical endometriosis features (glands and stroma). It is however important to note that glands and stroma are not always present in the later stages of endometriosis when reactive fibrosis becomes the main histological feature.

Our results are largely similar to previously published data with minor exceptions. The sensitivities of both methods in the detection of USL DE were marginally higher in our study (TVS 74% and MRI 94%) than values previously reported (TVS 67% [3], MRI 70–85% [3, 5, 20]) (Table 4). Aside from the selection bias, it is possible that is due to the improving skillset required for USL DE detection. The higher USL sensitivity observed might be also due to high association of USL and bowel endometriosis lesions, which was found in 21 out of 34 patients with affected USL (62%). The presence of bowel DE on TVS and MRI and significant site-specific tenderness on real-time TVS might have guided image readers to focus on the assessment of USL increasing the chance of identifying even smaller lesions. Additionally, the left USL is shorter than the right one due to the rotation and attachment of the sigmoid mesentery to the left pelvic side wall. Its close proximity to the bowel may explain the higher accuracy in DE detection on the left side when compared with the right side in our study (left USL: TVS 90%, MRI 90%; right USL: TVS 73%, MRI 76%). The slightly lower specificity in the detection of DE in USLs is due to 2 cases of negative histology in both right and left sides despite clear clinical and visual diagnosis (as mentioned above). Another factor to consider is generally difficult visualisation of USLs on the ultrasound, owing to their noncontrasting echogenicity and lack of standardised ultrasound technique. The latter was addressed in a recent communication by Leonardi et al. [21], suggesting a systematic structured approach to visualising USLs.

Vaginal DE was detected with lower sensitivity (TVS 55%, MRI 78%) than previously reported (Table 4). The possible reason for this is a new definition of vaginal lesions by IDEA consensus defining them as “a lesion located at the posterior and/or lateral vaginal fornix below the line passing along the caudal end of the peritoneum of the lower margin of the rectouterine peritoneal pouch, and above a line passing along the lower border of the posterior lip of the cervix” [8]. Although relatively easy for a sonographer or radiologist to distinguish, it is obvious that such complex anatomical definition might be difficult for a surgeon to apply when there is a limited clarity of anatomical involvement during a complex pelvic dissection, which is often the case when dealing with DE. This in turn led to increased rate of vaginal DE being reported intraoperatively by surgeons (reference standard) in contrast to index tests, which had much clearer view of structures involved. In contrast to the previously published data, we encountered no sigmoid lesions and no lower rectum lesions, but we regard the latter as acceptable since these lesions are very rare. Absence of sigmoid lesions however could be explained by the new IDEA of definition of bowel segment above the level of uterine fundus. The traditional topography of sigmoid loop as described in Gray's anatomy [22] “lying just to the left of the midline at the level of the third sacral body, where it bends inferiorly and is continuous with rectum,” might have contributed to higher rates of reporting of sigmoid lesions in the previous literature.

Although there were no statistically significant differences between MRI and TVS in the assessment of pelvic DE except for the higher accuracy of MRI in the assessment of USLs (*p*=0.04) and higher accuracy of TVS in the detection of POD obliteration (*p*=0.04), the dynamic aspect of ultrasound examination in addition to high-resolution transvaginal ultrasound probe appears to have resulted in higher specificity of ultrasound with 2.5% of false positive readings in comparison to MRI (6.4%) in overall pelvic DE detection. The disadvantage of ultrasound in the pelvic DE mapping is the challenging retrospective review of images with difficult second opinion, limited detection of extrapelvic lesions, and the lack of training and available expertise, which is the main reason for choosing MRI over ultrasound. It would be cost-effective for a busy endometriosis center to invest in the ultrasound training and then use MRI only as a second test in case of diagnostic uncertainty.

We cannot offer any formal validation of the IDEA consensus, given the limited number of participants, but we can report on the use in clinical practice and application to MRI and surgical reporting. The results in accuracy, similar to the previous research apart from the vagina and sigmoid colon, are suggestive that the new IDEA nomenclature does not have overall a negative impact on the detection rate. However, as mentioned above, some anatomical definitions might be challenging to apply in surgical assessment. It might be also beneficial if the formal IDEA proforma included magnetic resonance protocol covering the technical aspects, settings, and patient preparation in line with the current ESUR recommendations [9], hence simplifying it for use in daily practice. We also cannot comment on the accuracy of TVS and MRI and the use of the IDEA consensus in nonexpert hands outside endometriosis centers, since our results correspond only to advanced expertise appropriate for the centers.

To conclude, TVS and MRI show similar performance in pelvic DE mapping using the protocol based on the IDEA consensus and ESUR guidelines. Both diagnostic methods had the same accuracy in the detection of DE in the upper rectum, rectosigmoid, and ureter. TVS had marginally higher specificity but lower sensitivity in the assessment of bladder, USLs, vagina, rectovaginal septum, and pelvic DE overall.

## Figures and Tables

**Table 1:** Index tests methodology.

		Ultrasound	Magnetic resonance imaging
Technical parameters		Voluson E10 (GE medical Systems, Zipf, Austria)	3 T MR scanner with phased-array pelvic coil (Skyra, Siemens AG, Erlangen, Germany)
		Standard gynecology setting	Slice thickness 3-4 mm with interslice gaps 0.0 mm–1.0 mm

Preparation		No bowel preparation or contrast gel sonography	Fasting for 4 hours
			Butylscopolamine 1 mg i.v.

Technical protocol (standardised for use in every participant)		Transvaginal ultrasound (TV probe 7–9 MHz)	Protocol-part 1 for pelvic DE location
			(1) 2D T2W sequences in sagittal, axial and oblique plane^*∗*^
		Transabdominal ultrasound (curvilinear TA probe 4–7 MHz)	Protocol–part 2 for adnexal lesions
			(2) Dixon technique 2D (T1W images incl. with and without fat suppression sequences)^§^
			(3) DWI in axial plane^*∗∗*^
			(4) Postcontrast 2D T1W with fat suppression (i.v. gadolinium)^*∗∗*^

Imaged area	Pelvis	TVS	Whole pelvis from iliac crests to pubic bone
	Upper urinary tract	TAS	T2-weighted sequences in coronal plane from symphysis up to kidneys

^*∗*^ESUR recommends 2D T2W sequences for pelvic DE [9]. ^§^ESUR recommends 2D T1W sequences for endometriomas with Dixon technique as an alternative to confirm the presence of blood and to rule out a fat-containing lesion (such as dermoids) [9]. ^*∗∗*^ESUR recommended as optional sequences for ‘indeterminate' adnexal endometriosis (differential diagnosis of pelvic inflammatory disease, malignancy) [9]. 2D, two-dimensional; DE, deep endometriosis; DWI, diffusion-weighted imaging; ESUR, European Society of Urogenital Radiology guidelines [9]; MRI, magnetic resonance imaging; T1W, T1 weighted; T2W, T2 weighted; i.v. intravenous; TAS, transabdominal ultrasound; TVS, transvaginal ultrasound.

**Table 2:** Baseline characteristics.

Age (years), mean (SD)		32.4 (5.3)

BMI, mean (SD)		23.6 (3.9)

Parity, *n* (%)	Nulliparous	32 (62.3)
	Uniparous	13 (26.5)
	Multiparous	4 (8.2)

Fertility, *n* (%)	Primary infertility	21 (42.9)
	Secondary infertility	3 (6)
	Normal fertility	10 (20.4)
	No reproductive attempts	15 (31)

Hormonal treatment, *n* (%)	No treatment	33 (67.4)
	Progestogen only	12 (24.5)
	Combined oral contraception	4 (8.2)

Previous gynecological surgery investigating pain/infertility, *n* (%)	0	25 (51)
	1	20 (40.8)
	2	1 (2)
	3	3 (6)

Main symptoms, *n* (%)	Dysmenorrhea	41 (83.8)
	Dyspareunia	32 (62.3)
	Dyschesia	19 (38.8)
	Noncyclical pain	12 (24.5)
	Dysuria	8 (16.3)

Interval between initial symptoms experienced and diagnosis of endometriosis (years), mean (SD)		2.4 (1.9)

BMI, body mass index (kg/m^2^); *n*, number of participants; SD, standard deviation.

**Table 3:** Cross tabulation of study results.

Compartment	Areas investigated for DE	Index tests	TP (%)	FP (%)	TN (%)	FN (%)	Sensitivity (95% CI)	Specificity (95% CI)	PPV (95% CI)	NPV (95% CI)	LR+ (95% CI)	LR− (95% CI)	Accuracy (95% CI)	p-value
Anterior compartment	Bladder	TVS	8 (16.3%)	0 (0.0%)	40 (81.6%)	1 (2%)	0.89 (0.52–1.00)	1.00 (0.91–1.00)	1.00 (1.00–1.00)	0.98 (0.86–1.00)	Infinity	0.11 (0.02–0.71)	0.98 (0.89–1.00)	0.25
		MRI	9 (18.3%)	2 (4.1%)	38 (77.6%)	0 (0.0%)	1.00 (0.66–1.00)	0.95 (0.83–0.99)	0.82 (0.54–0.95)	1.00 (1.00–1.00)	20 (5.1–77.2)	0.00	0.96 (0.86–0.99)	
	Ureteric obstruction by DE	TVS/TAS	5 (10.2%)	0 (0.0%)	44 (89.8%)	0 (0.0%)	1.00 (0.48–1.0)	1.00 (0.92–1.00)	1.00 (1.00–1.00)	1.00 (1.00–1.00)	Infinity	0.00	1.00 (0.93–1.00)	1.000
		MRI	5 (10.2%)	0 (0.0%)	44 (89.8%)	0 (0.0%)	1.00 (0.48–1.0)	1.00 (0.92–1.0)	1.00 (1.00–1.00)	100 (1.00–1.00)	Infinity	0.00	1.00 (0.93–1.00)	

Posterior compartment	USL^§^	TVS	25 (51%)	5 (10.2%)	10 (20.4%)	9 (18.3%)	0.74 (0.56–0.87)	0.67 (0.38–0.88)	0.83 (0.70–0.91)	0.53 (0.36–0.68)	2.21 (1.05–4.64)	0.40 (0.20–0.77)	0.71 (0.57–0.83)	0.039
		MRI	32 (65.3%)	6 (12.2%)	9 (18.3%)	2 (4.1%)	0.94 (0.80–0.99)	0.60 (0.32–0.84)	0.84 (0.74–0.91)	0.82 (0.52–0.95)	2.35 (1.26–4.40)	0.10 (0.02–0.40)	0.84 (0.70–0.93)	
	Right USL	TVS	10 (20.4%)	5 (10.2%)	26 (53%)	8 (16.3%)	0.56 (0.31–0.78)	0.84 (0.66–0.95)	0.67 (0.45–0.83)	0.76 (0.65–0.85)	3.44 (1.40–8.50)	0.53 (0.31–0.91)	0.73 (0.59–0.85)	0.001
		MRI	17 (34.7%)	11 (22.4%)	20 (40.8%)	1 (2%)	0.94 (0.73–1.00)	0.65 (0.45–0.81)	0.61 (0.49–0.72)	0.95 (0.75–0.99)	2.66 (1.63–4.33)	0.09 (0.01–0.59)	0.76 (0.61–0.87)	
	Left USL	TVS	21 (42.9%)	0 (0.0%)	23 (46.9%)	5 (10.2%)	0.81 (0.61–0.93)	1.00 (0.85–1.00)	1.00 (1.00–1.00)	0.82 (0.68–0.91)	0.00	0.19 (0.09–0.42)	0.90 (0.78–0.97)	0.220
		MRI	23 (46.9%)	2 (4.1%)	21 (42.9%)	3 (6.1%)	0.88 (0.70–0.98)	0.91 (0.72–0.99)	0.92 (0.75–0.98)	0.88 (0.71–0.95)	10.17 (2.69–38.52)	0.13 (0.04–0.37)	0.90 (0.78–0.97)	
	Upper rectum	TVS	10 (20.4%)	0 (0.0%)	39 (79.6%)	0 (0.0%)	1.00 (0.69–1.00)	1.00 (0.91–1.00)	1.00 (1.00–1.00)	1.00 (1.00–1.00)	Infinity	0.00	1.00 (0.93–1.00)	1.000
		MRI	10 (20.4%)	0 (0.0%)	39 (79.6%)	0 (0.0%)	1.00 (0.66–0.89)	1.00 (0.91–1.00)	1.00 (1.00–1.00)	1.00 (1.00–1.00)	Infinity	0.00	1.00 (0.93–1.00)	
	Rectosigmoid	TVS	16 (32.7%)	5 (10.2%)	27 (55.1%)	1 (2%)	0.94 (0.71–1.00)	0.84 (0.67–0.95)	0.76 (0.59–0.88)	0.96 (0.80–0.99)	6.20 (2.67–13.59)	0.07 (0.01–0.47)	0.88 (0.75–0.95)	1.000
		MRI	16 (32.7%)	5 (10.2%)	27 (55.1%)	1 (2%)	0.94 (0.71–1.00)	0.84 (0.67–0.95)	0.76 (0.59–0.88)	0.96 (0.80–0.99)	6.20 (2.67–13.59)	0.07 (0.01–0.47)	0.88 (0.75–0.95)	
	RVS	TVS	4 (8.2%)	0 (0.0%)	43 (87.8%)	2 (4.1%)	0.67 (0.22–0.96)	1.00 (0.92–1.00)	1.00 (1.00–1.00)	0.96 (0.87–0.98)	Infinity	0.33 (0.11–1.03)	0.96 (0.86–1.00)	0.125
		MRI	5 (10.2%)	3 (6.1%)	40 (81.6%)	1 (2%)	0.83 (0.36–1.00)	0.93 (0.81–0.99)	0.63 (0.35–0.84)	0.98 (0.87–1.00)	11.9 (3.79–37.67)	0.18 (0.03–1.07)	0.92 (0.80–0.98)	
	Vagina	TVS	6 (12.2%)	0 (0.0%)	38 (77.6%)	5 (10.2%)	0.55 (0.23–0.83)	1.00 (0.91–1.00)	1.00 (1.00–1.00)	0.88 (0.80 –0.94)	Infinity	0.45 (0.24–0.87)	0.90 (0.78–0.97)	0.688
		MRI	8 (16.3%)	2 (4.1%)	36 (73.5%)	3 (6.1%)	0.73 (0.39–0.94)	0.95 (0.82–0.99)	0.80 (0.50–0.94)	92.31 (0.82–0.97)	13.82 (3.42–55.9)	0.29 (0.11–0.76)	0.90 (0.78–0.97)	
	POD obliteration	TVS	35 (71.5%)	1 (2%)	8 (16.3%)	5 (10.2%)	0.88 (0.73–0.96)	0.89 (0.52–1.00)	0.97 (0.85–1.00)	0.62 (0.41–0.79)	7.87 (1.24–50.16)	0.14 (0.06–0.33)	0.88 (0.75–0.95)	0.040
		MRI	23 (46.9%)	5 (10.2%)	4 (8.2%)	17 (34.7%)	0.58 (0.41–0.73)	0.44 (0.14–0.79)	0.82 (0.71–0.90)	0.19 (0.09–0.35)	1.03 (0.54–1.97)	0.96 (0.42–2.16)	0.55 (0.40–0.69)	

Pelvis	Overall pelvic DE^*∗∗*^	TVS	80 (20.4%)	10 (2.5%)	280 (71.4%)	22 (5.6%)	0.78 (0.69–0.86)	0.97 (0.94–0.98)	0.89 (0.81–0.94)	0.93 (0.90–0.95)	22.8 (12.30–42.20)	0.22 (0.15–0.32)	0.92 (0.89–0.94)	
		MRI	93 (23.7%)	25 (6.4%)	265 (67.6%)	9 (2.3%)	0.91 (0.84–0.96)	0.91 (0.88–0.94)	0.79 (0.72–0.84)	0.97 (0.94–0.98)	10.58 (7.24–15.46)	0.10 (0.05–0.18)	0.91 (0.88–0.94)	

§At least one affected side correctly identified. ^*∗∗*^Total DE lesions are the sum of all individual sites affected in all participants. CI, confidence intervals; DE, deep endometriosis; FN, false negative; FP, False positive; LR+, positive likelihood ratio; LR−, negative likelihood ratio; MRI, magnetic resonance imaging; NPV, negative predictive value; *p*-value, probability value of ultrasound vs. MRI performance (<0.05 considered significant); POD, pouch of Douglas; PPV, positive predictive value; RVS, rectovaginal septum; USL, uterosacral ligament; TAS, transabdominal ultrasound scan; TN, true negative TP, true positive; TVS, transvaginal ultrasound scan.

**Table 4:** Comparison of results with previous research.

Pelvis	Areas investigated for DE	Index tests	Sensitivity (%)		Specificity (%)	
			This study	Previous literature (author, year)	This study	Previous literature (author, year)
Anterior compartment	Bladder	TVS	89.0	62.0 (Guerriero, 2015) [4]	100.0	100 (Guerriero, 2015) [4]
				100 (Exacoustos, 2014) [12]		96.8 (Exacoustos, 2014) [12]
				44.0 (Savelli, 2009) [23]		100 (Savelli, 2009) [23]
		MRI	100.0	64.0 (Medeiros, 2015) [5]	95.0	98.0 (Medeiros, 2015) [5]
				81.0 (Krüger, 2013) [20]		94.7 (Krüger, 2013) [20]

Posterior compartment	USL	TVS	74.0	67.0 (Guerriero, 2018) [3]	67.0	86.0 (Guerriero, 2018) [3]
		MRI	94.0	70.0 (Guerriero, 2018) [3]	60.0	93.0 (Guerriero, 2018) [3]
				85.0 (Medeiros, 2015) [5]		80.0 (Medeiros, 2015) [5]
				77.5 (Krüger, 2013) [20]		68.2 (Krüger, 2013) [13]
	Right USL	TVS	56.0	80.7 (Exacoustos, 2014) [12]	84.0	87.2 (Exacoustos, 2014) [12]
		MRI	94.0	93.0 (Bazot, 2011) [24]	65.0	72.0 (Bazot, 2011) [24]
	Left USL	TVS	81.0	82.8 (Exacoustos, 2014) [12]	100.0	85.0 (Exacoustos, 2014) [12]
		MRI	88.0	89.0 (Bazot, 2011) [24]	91.0	61.0 (Bazot, 2011) [24]
	Rectum lower/upper	TVS	100.0	89.7^§^ (Exacoustos, 2014) [12]	100.0	86.2^§^ (Exacoustos, 2014) [12]
				94.4^*∗∗*^ (Exacoustos, 2014) [12]		84.9^*∗∗*^ (Exacoustos, 2014) [12]
		MRI	100.0	—	100.0	—
	Rectosigmoid	TVS	94.0	85.0 (Guerriero, 2018) [3]	84.0	96.0 (Guerriero, 2018) [3]
		MRI	94.0	85.0 (Guerriero, 2018) [3]	84.0	95.0 (Guerriero, 2018) [3]
				83.0 (Medeiros,2015) [5]		88.0 (Medeiros, 2015) [5]
				80.2 (Krüger, 2013) [20]		77.5 (Krüger, 2013) [20]
	RVS	TVS	67.0	59.0 (Guerriero, 2018) [3]	100.0	97.0 (Guerriero, 2018) [3]
				73.9 (Exacoustos, 2014) [12]		86.2 (Exacoustos, 2014) [12]
		MRI	83.0	66.0 (Guerriero, 2018) [3]	93.0	97.0 (Guerriero, 2018) [3]
				77.0 (Medeiros, 2015) [5]		95.0 (Medeiros, 2015) [5]
	Vagina	TVS	55.0	58.6 (Exacoustos, 2014) [12]	100.0	82.7 (Exacoustos, 2014) [12]
		MRI	73.0	82.0 (Medeiros, 2015) [5]	95.0	82.0 (Medeiros, 2015) [5]
				81.4 (Krüger, 2013) [20]		81.7 (Krüger, 2013) [20]

^§^Cranial (upper) rectum. ^*∗∗*^Lower (caudal) rectum. DE, deep endometriosis; USL, uterosacral ligaments; RVS, rectovaginal septum; TVS, transvaginal ultrasound; MRI, magnetic resonance imaging.

## Data Availability

This study was a single-unit study, where data were recorded on preprinted forms as a part or preoperative and intraoperative reporting. Patients' data were anonymised in the final database, which was sent to the statistician as an excel sheet. The descriptive summary of cases (number of true positives, negatives, etc.) is included within the article. All data and information used in the introduction and discussion are included in the references and are available online.

## Supplementary Materials

Supplementary Materials Supplementary Table 1: mapping of endometriosis using modified IDEA protocol DE, deep endometriosis; IDEA, International Deep Endometriosis Analysis; MRI, magnetic resonance imaging; POD, pouch of Douglas; T2W, T2-weighted. §Kissing ovaries on transverse plane are visualised as the ovaries adherent to each other, usually behind the uterus, corresponding to dense inter-ovarian adhesions and frozen pelvis. Supplementary Table 2: IDEA anatomical definitions. USL, uterosacral ligaments. ^*∗*^IDEA protocol does not offer anatomical definition of USL but comments on methodology of USLs evaluation and ultrasound appearance when USLs are affected by DE [7].
