# Supplementary material for: Diagnostic Accuracy of Ultrasound and MRI in the Mapping of Deep Pelvic Endometriosis Using the International Deep Endometriosis Analysis (IDEA) Consensus
Source: Biomed Res Int. 2020 Jan 30;2020:3583989. doi: 10.1155/2020/3583989 (PMC7011347; doi:10.1155/2020/3583989)
Supplement: Supplementary Materials — Supplementary Table 1: mapping of endometriosis using modified IDEA protocol DE, deep endometriosis; IDEA, International Deep Endometriosis Analysis; MRI, magnetic resonance imaging; POD, pouch of Douglas; T2W, T2-weighted. §Kissing ovaries on transverse plane are visualised as the ovaries adherent to each other, usually behind the uterus, corresponding to dense inter-ovarian adhesions and frozen pelvis. Supplementary Table 2: IDEA anatomical definitions. USL, uterosacral ligaments. ∗IDEA protocol does not offer anatomical definition of USL but comments on methodology of USLs evaluation and ultrasound appearance when USLs are affected by DE [7]. [file 3583989.f1.docx]

|  | | **Ultrasound** | **MRI** | **Laparoscopy** |
| --- | --- | --- | --- | --- |
| **A. Assessment of the uterus and adnexa** | | | | |
| 1st step | Uterus, adnexa | Routine evaluation of the uterus, fallopian tubes and ovaries, including presence of adenomyosis and endometriomas | Routine evaluation of the uterus, Fallopian tubes and ovaries, including presence of adenomyosis and endometriomas | Routine evaluation of the uterus, Fallopian tubes and ovaries |
| 2nd step | Soft markers / Adhesions | Evaluation of uterine and ovarian mobility using soft markers (‘sliding sign‘ and site-specific tenderness) | Evaluation of signs of adhesions (**1)spiculated hypointense peritoneal strands (T2W sequences), (2) tethering of pelvic structures (3) distorted anatomy from adhesions (posterior displacement of uterus and ovaries, hydrosalpinx, loculated fluid collections and others).** | Evaluation of adhesions, superficial endometriosis |
| **B. Assessment of the anterior and posterior compartment** | | | | |
| 3rd step | Obliteration | Assessment of the anterior (uterovesical region) and posterior (POD) compartment obliteration using 'sliding sign' of bladder or anterior bowel against uterus. | Assessment of the anterior and posterior compartment (POD) obliteration using combination of signs : **anterior or posterior displacement of uterus, tethered appearance of bowel / bladder in direction of uterus, fibrotic plaques or nodules in bladder/bowel/USLs/between uterus and bowel or bladder, the presence of kissing ovaries§ and others.** | Assessment of the anterior and posterior compartment obliteration |
| 4rd step | Deep endometriosis | DE nodules in the anterior compartment (bladder, pelvic part of ureters) | DE nodules in the anterior compartment (bladder, pelvic part of ureters) | DE nodules in the anterior compartment (bladder, pelvic part of ureters) |
|  |  | DE nodules in the posterior compartment (uterosacral ligaments, vagina, rectovaginal septum, rectum, rectosigmoid and sigmoid). | DE nodules in the posterior compartment (uterosacral ligaments, vagina, rectovaginal septum, rectum, rectosigmoid and sigmoid) | DE nodules in the posterior compartment (uterosacral ligaments, vagina, rectovaginal septum, rectum, rectosigmoid and sigmoid) |
| **C. Assessment of upper urinary tract in all women with DE** | | | | |
| 5th step | Presence and grading of hydronephrosis | Assessment of the upper urinary tract using transabdominal ultrasound | Assessment of the urinary tract using T2W sequences in coronal plane with field of view extended to upper pole of kidneys | Visual inspection of the visible course of ureters, dissection where indicated by the imaging |

**Supplementary table 1. Mapping of endometriosis using modified IDEA protocol**

DE, deep endometriosis; IDEA, International Deep Endometriosis Analysis; MRI, magnetic resonance imaging; POD, pouch of Douglas; T2W, T2-weighted.

§Kissing ovaries on transverse plane are visualised as the ovaries adherent to each other, usually behind the uterus, corresponding to dense inter-ovarian adhesions and frozen pelvis.

| **Location of deep endometriosis** | **Definition** |
| --- | --- |
| Rectovaginal septum | Rectovaginal space below the line passing along the lower border of the posterior lip of the cervix |
| Lower (retroperitoneal) anterior rectum | Bowel segment below the insertion of uterosacral ligaments on the cervix |
| Upper anterior rectum | Bowel segment above the uterosacral ligaments |
| Rectosigmoid junction | Bowel segment at the level of the uterine fundus |
| Anterior sigmoid | Bowel segment above the level of the uterine fundus |
| Vaginal wall | Rectovaginal space below the line passing along the caudal end of the peritoneum of the lower margin of the rectouterine peritoneal pouch and above the line passing along the lower border of the posterior lip of the cervix |
| Uterosacral ligaments (USL) | Collection of connective tissue that originate from the posterolateral aspect of the cervix and vaginal fornix and inserted to lateral aspect of rectum and front of sacrum*. |
| Bladder | Bladder is divided into four zones:   1. Trigonal zone - within 3 cm of the urethral opening, smooth triangular area defined by the urethral and ureteral orifices 2. Base - portion adjacent to upper anterior vagina and the supravaginal portion of the cervix 3. Dome - intra-abdominal portion superior to the base 4. Extra-abdominal bladder (retroperitoneal) |

Supplementary table 2. IDEA anatomical definitions. USL, uterosacral ligaments. *IDEA protocol doesn't offer anatomical definition of USL but comments on methodology of USLs evaluation and ultrasound appearance when USLs are affected by DE [7].
